# Academic pediatric clinical research: factors associated with study implementation duration

**DOI:** 10.1186/s12874-016-0138-y

**Published:** 2016-03-29

**Authors:** Delphine Meier-Girard, Annick Tibi, Hendy Abdoul, Sonia Prot-Labarthe, Françoise Brion, Olivier Bourdon, Corinne Alberti

**Affiliations:** Université Paris Diderot, Sorbonne Paris Cité, UMR-1123 ECEVE, F-75019 Paris, France; AP-HP, Hôpital Robert Debré, Unité d’Epidémiologie Clinique, F-75019 Paris, France; Inserm, U1123 and CICEC 1426, F-75019 Paris, France; University of Basel, University Children’s Hospital (UKBB), Basel, Switzerland; Université Paris Descartes, Sorbonne Paris Cité, F-75270 Paris, France; Département d’Essais Cliniques, AP-HP, Agence Générale des Equipements et Produits de Santé, F-75013 Paris, France; AP-HP, Hôpital Robert Debré, Pharmacie à Usage Intérieur, F-75019 Paris, France; Department of Paediatric Pulmonology, University Children`s Hospital Basel (UKBB), Spitalstrasse 33, 4056 Basel, Switzerland

**Keywords:** Pediatrics, Biomedical research, Academic medical centers, Delay in studies implementation

## Abstract

**Background:**

The ethical, methodological, and technical aspects of pediatric research, often results in complications and delays in implementation. Our objective was to identify factors associated with the implementation duration of hospital-based pediatric studies.

**Methods:**

All hospital-based pediatric studies sponsored by AP-HP between 2002 and 2008 were retrospectively identified. Association of the funding mechanism and methodological factors with the implementation duration was assessed using a multivariable mixed linear model. Pharmaceutical factors were explored as part of a subgroup analysis restricted to the studies involving drug therapy. Given that we took an exploratory approach, factors associated with implementation duration with *p* < 0.10 were kept in the final models.

**Results:**

A total of 139 studies were evaluated. The median implementation duration was 17.1 months (range: 0.9-55.3 months), and tended to increase over time (from 14.9 [25^th^ percentile-75^th^ percentile: 11.5-19.9] months in 2002 to 23.7 [15.2-31.0] months in 2008, *p* = 0.01). External (coefficient [95 % confidence interval]: -7.7 [-11.9;-3.5] months, *p* < 0.001) and internal funding (-5.3 _95 % CI_ [-9.8;-0.8], *p* = 0.02) compared to governmental funding and number of centers (-0.1 _95 % CI_[-0.2;0.02] months for 1 center increase, *p* = 0.07) were associated with reduced duration, whereas interventional study (either involving drug therapy (6.0 _95 % CI_[0.7;11.3] months, *p* = 0.03 or not (3.5 _95 % CI_[-0.3;7.3] months, *p* = 0.06) was associated with increased duration compared to observational study. Regarding the 35 studies involving drug therapy, external funding decreased duration (-6.7 _95 % CI_[-13.2;-0.2] months, *p* = 0.05), whereas studies involving solely a pediatric population (7.8 _95 % CI_[1.1;14.5] months, *p* = 0.01) (compared to mixed adult-pediatric population), a placebo-controlled design (6.6 _95 % CI_[0.9;12.3] months, *p* = 0.01), and inappropriate drug formulation for at least one drug used in the study (6.9 _95 % CI_[-0.2;14.0] months, *p* = 0.06) were associated with increased duration.

**Conclusion:**

Implementation of hospital-based pediatric studies primarily faced delays when they were interventional and, in particular, when they involved drug therapy. Regarding the latter, difficulties that resulted in delayed studies arose with respect to the supply of drugs and placebo in age-appropriate dosages and route of administration. Therefore, difficulties related to the use of pharmaceuticals need to be anticipated earlier in order to avoid implementation delays.

**Electronic supplementary material:**

The online version of this article (doi:10.1186/s12874-016-0138-y) contains supplementary material, which is available to authorized users.

## Background

Pediatric research faces specific ethical, methodological, and technical obstacles [1–4]. Moreover, the lack of financial rewards for the pharmaceutical industry is an additional obstacle for pediatric clinical trials that involve drug therapy [5, 6]. Those obstacles are well recognized and result in a lack of pediatric clinical studies [7, 8]. To address the paucity of pediatric research and to encourage investment by pharmaceutical companies, the United States and Europe enacted new legislations around efficacy and safety of drug trials in children [9–16]. Those legislations have led to significant improvements in patient safety, trial validity, data reliability, and has addressed the availability of drugs with age-appropriate information [17, 18]. However, the overall impact on the number of clinical trials performed remains modest [8, 19], and only a moderate correlation exists between the clinical trial activity and the pediatric burden of disease [20]. Furthermore, it has been shown that drugs frequently used in pediatric patients were underrepresented among drugs qualifying for pediatric exclusivity [17, 21–23]. Therefore, in addition to industry-driven research, hospital-based investigation of pediatric drug therapy is needed [23, 24].

However, previous studies identified the heavy regulatory burden [5, 25–28], the pharmaceutical issues and difficulties in establishing contracts with pharmaceutical companies [25, 26, 29–32], the inadequate training of investigators, difficulty functioning as both clinician and investigator [5, 25, 27, 33–37], as well as suboptimal communication across disciplines involved [25], as the main obstacles in conducting academic pediatric studies. Further obstacles relate to the complexity of the 2001 European directive and variability in its interpretation across European Union countries which have resulted in further delays in conducting trials, and has contributed to the loss of European competitiveness in academic research over the past decade [24, 38]. Delays affect the competitiveness and attractiveness of European clinical research, and may also be deleterious for the patients, which dissuades some medical doctors from participation in clinical trials [5, 25, 26, 33]. In July, 2012, the European Commission finalized a proposal for a Clinical Trial Regulation, which would replace the 2001 European directive [39]. The regulation aims at facilitating the implementation of clinical trials and reducing the regulatory burden via a risk-proportionate approach. Thus, it should result in the reduction of both duration and cost for trial implementation. In the context of the ongoing discussion on the European Clinical Trial Regulation, it is of interest to quantify the factors involved in delayed trial implementation. It should allow ultimately for an improved understanding of the obstacles faced by research stakeholders (e.g. investigators, pharmacists, methodologists, and sponsor representatives), the facilitation of collaboration across involved disciplines, and the development of preventive strategies.

The objective of this study was to identify factors associated with the implementation duration of hospital-based pediatric studies.

## Methods

### Ethics statement

This study did not involve any patients and, therefore, required neither written consent nor information sheet. The Institutional Review Board of the Paris North Hospitals, Paris 7 University, Paris Public Hospital Network (AP-HP), approved the study protocol (N° 12-049).

### Selection of studies

All hospital-based pediatric studies sponsored by the Assistance publique – Hôpitaux de Paris (AP-HP) between January 1, 2002 and December 31, 2008 were selected. AP-HP is the public hospital system of the city of Paris and its suburbs. An additional file describes the French hospital-based Biomedical Research System, and the AP-HP division in more detail (Additional file 1). Studies were retrieved from the AP-HP sponsor’s database (recording all AP-HP-sponsored studies) and from the AP-HP central pharmacy’s database (recording all studies AP-HP-sponsored and involving drug therapy). The AP-HP central pharmacy is in charge of the pharmaceutical process for AP-HP-sponsored biomedical research.

### Data collection

This study is part of a larger project aimed at understanding the issues faced by all stakeholders during a study’s implementation. Data were collected between 2009 and 2010. We first conducted a qualitative study on the perceptions and experiences of healthcare professionals involved in pediatric hospital-based research (principal investigators, pharmacists, sponsor representatives, French drug agency representatives) [25]. The current study was based on the knowledge and understanding gained from this qualitative analysis.

Data were collected using a web-based Case Report Form system specifically developed for the study (Janus, PHP) from the AP-HP sponsor’s database, the AP-HP central pharmacy’s database, and the studies’ protocols. Missing data were sought in the ClinicalTrials.gov registry [40], and publications related to the studies. Ultimately, the involved Clinical Trial Units and investigators were asked to provide the remainder of missing data.

Collected data included information on funding and study methodology. Pharmaceutical information was collected for the studies involving drug therapy. Data related to funding included the date of grant approval and the funding mechanism (governmental funding, AP-HP internal funding, or external funding). External funding corresponds to funding from pharmaceutical companies, associations, or foundations. Data related to the methodology were: type of the coordinating center (pediatric hospital, general hospital), number of centers, whether the study was international or national, study type (interventional involving drug therapy, interventional without drug therapy, observational), characteristics of the disease (whether the disease was rare and/or chronic), study population (pediatric population only, or mixed population, *i.e.,* including children and adults), number of patients to include, and participation duration of the subjects. Data related to the pharmaceutical aspects were: randomization, blinded patient, blinded investigator, duration of treatment, control group (standard treatment, placebo*,* no control group), whether the drug therapy was already approved in a pediatric population, route of administration (enteral, parenteral), and whether the formulation of the available drug therapy was in an age-appropriate dosage and route of administration (appropriate, intermediate, inappropriate). The formulation was defined as intermediate when only capsules were available for the study, though children under six years, who were unable to swallow capsules, were included. In this case, the capsules must be opened to collect the powder which is then incorporated into an aliment or a fluid to be administrated (except for delayed-release and protective-coated capsules). The formulation was also defined as intermediate when an injectable form was utilized for enteral administration. The formulation was defined as inappropriate when only tablets were available for the study, even though children under 6 years were included. In this situation, the tablets must be crushed to get a powder, which then requires an additional pharmaceutical medium to ensure that the pharmacological properties and the pharmacokinetics of the drug therapy are not altered.

### Statistical analysis

Results are expressed as numbers and percentages for categorical variables and as a mean (standard deviation) or median [25^th^ percentile;75^th^ percentile] for continuous variables, according to their distribution.

The implementation duration was defined as the time between the date of grant and the inclusion of the first patient (i.e. date of first informed consent). Probability over time to include the first patient was depicted using the inverse Kaplan-Meier curve. Differences in distribution of implementation duration according to the funding mechanism and the methodological factors were assessed using Pearson’s (or Spearman’s, as appropriate) correlation coefficient for quantitative variables and *t*-test (or Wilcoxon test, as appropriate) for categorical variables. For comparison of the mean value of the implementation duration across more than two groups, one-way ANOVA was performed. Independent prognostic factors of implementation duration were assessed using a multivariable mixed linear model, with the random effect of the year of grant [41]. A random effect was included to adjust for potential residual clustering of data within the year of the grant. Pharmaceutical factors were explored as part of a subgroup analysis restricted to the studies involving drug therapy. For all multivariable regression models, *p* < 0.20 was used for the variable entry criteria. Then, a stepwise selection procedure was applied. Given that the statistical analyses were essentially exploratory, factors associated with the considered outcome with *p* < 0.10 were kept in the final model. Variables dropped at earlier stages were re-evaluated for inclusion in the final model. Normality of residuals and absence of heteroscedasticity were checked.

All tests were two-sided. Statistical analysis was performed using the R package Version 2.10 [42] and SAS 9.2 software (SAS Institute, Cary, NC, USA).

## Results

### Description of the studies

We identified 145 pediatric studies sponsored by the AP-HP between 2002 and 2008. Six were excluded since they stopped before the first inclusion (Table 1). Among them, 2 were observational studies (33 %), 3 were interventional studies without drug therapy (50 %), and 1 was an interventional study involving drug therapy (17 %). A total of 139 studies were analyzed. Seventy-two (52 %) were observational studies, 32 (23 %) were interventional without drug therapy, and 35 (25 %) were interventional involving drug therapy (Table 1).

**Table 1:** Characteristics of the hospital-based pediatric studies sponsored by the Assistance Publique-Hôpitaux de Paris between 2002 and 2008

Characteristics	Observational studies (*n* = 72)	Interventional studies without drug therapy (*n* = 32)	Interventional studies involving drug therapy (*n* = 35)	*p*-value	Excluded studies (*n* = 6)	
						*NA*
Funding mechanism				0.07		*5*
Government	39 (54)	20 (63)	12 (34)		1 (100)	
External	20 (28)	4 (13)	14 (40)		0 (0)	
Internal	13 (18)	8 (25)	9 (26)		0 (0)	
Year of grant				-		*0*
2002	17 (24)	1 (3)	3 (9)		1 (17)	
2003	5 (7)	2 (6)	3 (9)		4 (67)	
2004	9 (13)	2 (6)	5 (14)		1 (17)	
2005	15 (21)	1 (3)	7 (20)		0	
2006	6 (8)	5 (16)	6 (17)		0	
2007	12 (17)	12 (38)	9 (26)		0	
2008	8 (11)	9 (28)	2 (6)		0	
Coordinator center				0.31		*0*
Pediatric hospital	28 (39)	15 (47)	19 (54)		6 (100)	
General hospital	44 (61)	17 (53)	16 (46)		0 (0)	
Number of centers	6 [2;16]	5 [2;16]	3 [1;15]	0.78	1 [1;1]	*1*
Monocentric study	12 (17)	5 (15.6)	14 (40)	0.01	4 (80)	*1*
International study	1 (1)	0 (0)	4 (11)	0.006	0 (0)	*0*
Rare disease	50 (69)	20 (63)	27 (77)	0.43	4 (67)	*0*
Chronic disease	60 (83)	22 (69)	26 (74)	0.22	4 (67)	*0*
Study population				0.003		*0*
Pediatric	30 (42)	19 (59)	27 (77)		6 (100)	
Mixed (children and adults)	42 (58)	13 (41)	8 (23)		0 (0)	
Number of patients to include	150 [82;388]	128 [66;240]	60 [36;100]	0.12	20 [20;25]	*3*
Length of participation, months	0.2 [0.03;12]	4 [0.2;17]	8 [0.5;24]	0.61	0.1 [0.03;2]	*2*
Referenced in ClinicalTrials.gov	21 (29)	32 (100)	24 (69)	<0.001	1 (17)	*0*

Values shown are median [25^th^ percentile; 75^th^ percentile] and number (percentage)

The studies mainly focused on rare and chronic diseases. There was no particular trend regarding the number of studies granted per year. The main source of funding in observational studies and in interventional studies without drug therapy was governmental (*n* = 39/72, 54 % and *n* = 20/32, 63 % respectively), whereas studies involving drug therapy more often involved external funding (*n* = 14/35, 40 %). There were more monocentric studies among studies involving drug therapy (*p* = 0.01), and studies involving drug therapy almost exclusively included a pediatric population (*p* = 0.003).

### Exploration of factors associated with study implementation duration

**Fig. 1:**
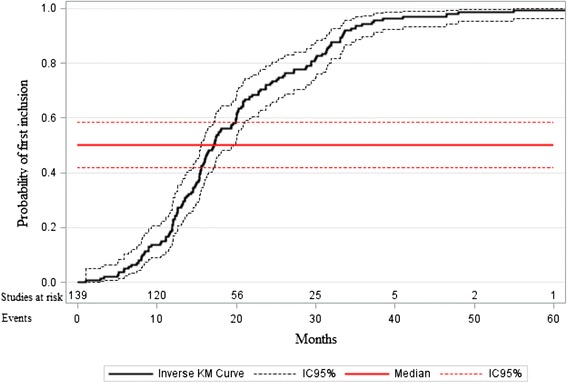
Probability over time to include the first patient

Figure 1 shows the probability over time to include the first patient (the confidence interval of the probability appears as a gray cloud). The median implementation duration was 17.1 months (95 % CI [15.6;19.9] months), ranging from 0.9 to 55.3 months. The univariate analysis of factors determining the implementation duration is presented in Table 2. The funding mechanism, type of study, and rare disease were associated with the implementation duration. The implementation duration tended to increase over time (*p* = 0.01).

**Table 2:** Bivariate analyses of factors associated with the duration of the study implementation: a- all included studies (*n* = 139), b- restricted to studies involving drug therapy (*n* = 35)

	*a-All studies (n = 139)*		*b-Studies involving drug therapy (n = 35)*	
Characteristics	Implementation duration in months, or correlation coefficient	*p*-value	Implementation duration in months, or correlation coefficient	*p*-value
*Grant characteristics*				
Funding mechanism		0.002		0.007
Government	22.6 (11.3)		25.2 [21.2;31.7]	
External	15.3 (9.5)		13.7 [11.9;17.3]	
Internal	18.9 (8.9)		18.0 [15.9;31.9]	
Year of grant		0.01		0.13
2002	14.9 [11.5;19.9]		17.8 [16.0;32.9]	
2003	19.1 [13.1;30.5]		22.8 [14.8;28.0]	
2004	17.4 [12.5;22.4]		15.9 [15.2;17.4]	
2005	12.3 [10.8;16.6]		15.9 [12.9;17.1]	
2006	20.8 [14.5;27.3]		22.1 [14.5;27.0]	
2007	20.9 [15.5;30.0]		21.7 [14.0;29.4]	
2008	23.7 [15.2;31.0]		36.5 [34.2;38.7]	
*Methodological characteristics*				
Coordinator center		0.85		0.46
General hospital	19.7 (10.9)		17.2 [13.7;24.4]	
Pediatric hospital	20.0 (10.7)		19.4 [15.1;29.6]	
Number of centers	*ρ* = -0.09	0.32	*ρ* = 0.09	0.62
Type of study		0.03		-
Interventional involving drug therapy	17.8 [13.9;28.3]		-	
Interventional without drug therapy	20.4 [14.8;30.1]		-	
Observational	15.5 [11.0;21.3]		-	
International study		0.81		0.77
Yes	21.7 [13.8;22.8]		22.2 [19.7;25.1]	
No	16.5 [12.7;24.3]		17.2 [14.1;30.8]	
Rare disease		0.007		0.30
Yes	21.1 (12.0)		19.4 [13.9;30.4]	
No	16.8 (6.4)		16.6 [15.2;18.1]	
Chronic disease		0.65		0.31
Yes	19.6 (10.3)		19.7 [14.1;29.4]	
No	20.7 (12.3)		15.9 [13.3;17.4]	
Study population		0.34		0.05
Pediatric population	20.7 (10.7)		19.4 [15.9;29.4]	
Mixed population	18.9 (10.9)		13.9 [10.6;17.2]	
Length of participation	*ρ* = 0.08	0.36	*ρ* = 0.12	0.49
*Pharmaceutical characteristics*				
Inclusion of children under 6 years old		-		0.24
Yes	-		15.9 [13.4;18.7]	
No	-		18.7 [14.2;29.9]	
Randomized study		-		0.04
Yes	-		20.8 [15.4;30.8]	
No	-		14.3 [11.9;17.4]	
Duration of treatment, days	-	-	*ρ* = 0.04	0.81
Blinded investigator and/or blinded patient		-		0.06
Yes	-		23.0 [15.9;31.3]	
No	-		16.4 [12.3;21.1]	
Placebo-controlled study		-		0.01
Yes	-		29.4 [17.4;31.9]	
No	-		16.4 [12.3;19.9]	
At least a drug of the study not approved in pediatric population		-		0.33
Yes	-		17.4 [14.2;27.8]	
No	-		22.9 [16.3;28.6]	
Age-appropriate formulation		-		0.05
Appropriate	-		16.7 [13.6;25.3]	
Intermediate at least for one drug of the study	-		17.4 [17.1;18.7]	
Inappropriate at least for one drug of the study	-		30.7 [24.6;32.8]	
Route of administration		-		0.37
Enterally at least for one drug of the study	-		20.0 [14.8;30.4]	
Parenterally	-		17.6 [13.5;21.9]	

Values of the duration of the study implementation are reported as mean (Standard Deviation) or median [25^th^ percentile;75^th^ percentile]

The multivariable mixed linear model analysis showed that government funding and interventional studies, both involving or without drug therapy, were significantly associated with delayed implementation, and an increasingly number of centers tended to be associated (Table 3).

**Table 3:** Analysis of factors associated with the implementation duration using multivariable regression: a- all included studies (*n* = 139), b- restricted to studies involving drug therapy (*n* = 35)

**a) All studies, multivariable mixed linear model with a random intercept for year of grant (** ***n*** **= 139)**		
** Variable**	**Coefficient (95 % CI)**	***p*** **-value**
Intercept	22.3 (18.5;26.1)	
** Funding mechanism** (ref. = Government)		**<0.001**
External	-7.7 (-11.9;-3.5)	*<0.001*
Internal	-5.3 (-9.8;-0.8)	*0.02*
** Number of centers** ^a^	-0.1 (-0.2;0.02)	**0.07**
** Type of study** (ref = Observational)		**0.003**
Interventional involving drug therapy	6.0 (0.7;11.3)	*0.03*
Interventional without drug therapy	3.5 (-0.3;7.3)	*0.06*
**b) Studies involving drug therapy, using multivariable linear regression (** ***n*** **= 35)**		
** Variable**	**Coefficient (95 % CI)**	***p*** **-value**
Intercept	14.9 (6.9;22.9)	
** Funding mechanism** (ref. = Government)		**0.08**
External	-6.7 (-13.2;-0.2)	*0.05*
Internal	-2.9 (-9.8;4.0)	*0.41*
** Study population** (ref. = Mixed population)		**0.01**
Pediatric population	7.8 (1.1;14.5)	
** Placebo-controlled study**	6.6 (0.9;12.3)	**0.01**
** Age-appropriate formulation** (ref. = Appropriate)		**0.05**
Intermediate at least for one drug of the study	-3.6 (-13.0;5.8)	*0.44*
Inappropriate at least for one drug of the study	6.9 (-0.2;14.0)	*0.06*

^a^For an increase of 1 centre

For all multivariable regression models, *P* < 0.20 was used for the variable entry criteria. Then, a stepwise selection procedure was applied. Because the statistical analyses were essentially exploratory, factors associated with the considered outcome with *P* < 0.10 were kept in the final model. Variables dropped at earlier stages were re-evaluated for inclusion in the final model

Values shown are coefficient, 95 % confidence interval (95 % CI). Individual *p*-value for each category of the variables are shown (in italics), as well as the global *p*-value of the variable (in bold)

### Exploration of factors associated with implementation duration of studies involving drug therapy

The pharmaceuticals characteristics of the 35 studies involving drug therapy are shown in Table 4. Studies were mainly randomized (*n* = 26, 74 %), 16 (46 %) involved blinded investigators, 17 (49 %) blinded patients, 13 (37 %) were placebo controlled, and 9 (26 %) involved a drug therapy with an intermediate or inappropriate formulation with respect to the age of the participants (*n* = 3, 9 % and *n* = 6, 17 % respectively).

**Table 4:** Pharmaceuticals characteristics of studies involving drug therapy (*n* = 35)

Pharmaceutical characteristics	Studies involving drug therapy (*n* = 35)
Inclusion of children under 6 years old	26 (74)
Randomized study	26 (74)
Duration of treatment, days	60 [5;365]
Blinded investigator	16 (46)
Blinded patient	17 (49)
Control group	
Standard treatment	10 (29)
Placebo	13 (37)
No control group	12 (34)
At least a drug of the study not approved in pediatric population	10 (33)
Age-appropriate formulation	
Appropriate	26 (74)
Intermediate at least for one drug of the study	3 (9)
Inappropriate at least for one drug of the study	6 (17)
Route of administration	
Enterally at least for one drug of the study	19 (54)
Parenterally	16 (46)

Values shown are median [25^th^ percentile; 75^th^ percentile] and number (percentage)

The univariate analysis of factors determining the implementation duration of studies involving drug therapy is presented in Table 2. The funding mechanism, study population, randomization, blinded patient and/or investigator, placebo-controlled study status, and drug formulation were associated with implementation duration.

The multivariable mixed linear model analysis showed that an exclusively pediatric population, placebo-controlled study, and inappropriate drug formulation were significantly associated with delayed implementation for the studies involving drug therapy (Table 3). Government funding tended to be associated with delayed implementation.

## Discussion

### Main results

Our findings show that implementation duration increased over time, with large fluctuations (ranging from 1 to 55 months) depending on the funding mechanism, number of centers involved, and type of study. There was no particular trend regarding the number of studies granted per year.

Government funding was associated with delayed implementation as compared to external (-7.7 _95 % CI_[-11.9;-3.5] months, *p* < 0.001) and internal funding (-5.3 _95 % CI_[-9.8;-0.8] months, *p* = 0.02). One main explanation was that the time needed to mobilize resources is longer for government funding than for the other types of funding. Indeed, while there is a specific process that must be undertaken in order to mobilize resources from the government, money dedicated to internal funding is held by the AP-HP sponsor and can be immediately mobilized. Similarly, external funding from pharmaceutical companies, associations, and foundations can be rapidly mobilized. Furthermore, internal funding provides a lower overall amount of funding (≤100,000 euros) than does government funding. It is, therefore, dedicated in principle to smaller studies, and the regulatory process is less complicated than for government funding. This may also explain why studies with internal funding started earlier than studies with government funding. Finally, the benefits of sharing expertise, resources and competences in the context of external funding might reduce implementation duration as compared to studies with government funding [12, 43, 44].

Interventional studies started later than observational studies, which is consistent with the heavier regulatory requirements to meet before the first inclusion in such studies. Interventional studies involving drug therapy were more delayed (6.0 ± 2.7 months, *p* = 0.03) than interventional studies without drug therapy (3.5 ± 1.9 months, *p* = 0.06). It would be of interest to future studies to compare the complexity and issues faced by the different stakeholders when implementing interventional studies with and without drug therapy.

Multi-center studies tended to include the first patient quicker than single center studies. Indeed, increasing the number of centers increases recruitment capacity, and thus the probability to include the first patient much earlier. However, involving many sites also results in a complex study set-up. Therefore, the number of patients and centers to involve should be considered carefully and in a well-balanced manner.

With regard to the subgroup of studies involving drug therapy, our findings again showed that government funding was associated with delayed implementation as compared to external funding (-6.7 ± 3.2 months, *p* = 0.05). Among the 35 studies involving drug therapy, 40 % had an external funding source. Given that external funding in studies involving drug therapy typically comes from pharmaceutical companies, this finding might reflect the benefit of public-private partnerships. The benefit of public-private partnerships seems even more apparent since there is no significant difference in the implementation duration between studies with government funding and studies with internal funding (-2.9 ± 3.4 months, *p* = 0.41) in this subgroup of studies.

Finally, pharmaceutical factors determining the implementation duration were identified. Age-inappropriate drug formulation and placebo-controlled studies significantly increased the implementation duration (respectively 6.9 ± 3.5 months, *p* = 0.06 and 6.6 ± 2.8 months, *p* = 0.01). Looking into the order history of these studies and/or meeting the stakeholders, we discovered that these studies had faced such issues as unexpected placebo, or problems with the supply of an age-appropriate drug formulation. In these situations, drug formulation development was instead carried out by hospital pharmacies or subcontractors, which resulted in major delays and supplementary costs.

### Strengths and weaknesses of the study (internal validity)

This study is the first to quantify the implementation duration of hospital-based pediatric clinical studies and to identify factors independently related to this duration. A limitation of the study is that the data were collected in 2009, which prevented us from describing more recent data. However, the issues highlighted by our study are not specific to this time period and are still relevant today. Although it is a retrospective study, there were no missing data and the sources of data were reliable. It would have been of interest to analyze the association between the risk level associated to the studies and their implementation duration; however, this data was not available. Nevertheless, the type of study closely reflects this information since the interventional pediatric studies are usually classified as high risk and the observational studies as low risk. Indeed, the regulations are much more stringent for pediatric populations compared to adult populations [25].

### Strengths and weaknesses of the study compared to other studies (external validity)

We focused on AP-HP-sponsored studies, and therefore on a single geographic region (the Paris conurbation), which may limit the external validity of our findings. However, the AP-HP is the leading academic sponsor in France, and the leading research center in Europe [45]. Moreover, the issues highlighted by our study are not specific to the AP-HP institution.

Academic research faces several obstacles for the implementation of their studies [5, 33], which result in implementation delays that have been quantified in our study.

A major issue in the implementation of studies is the regulatory burden. It is widely described in the literature [5, 46, 47] and highlighted in a qualitative study previously conducted by our group [25]. The present study shows that while there was no particular trend regarding the number of studies granted per year, the implementation duration increased over time. The increasing implementation duration over time might be related to an increasing regulatory burden. Osuntokun described this increasing regulatory burden over time, and discussed the difficulty for academic structures to apply the pediatrics regulations [5]. Indeed, these regulations are specifically targeted at pharmaceutical companies, and slow down academic research [24, 38]. The upcoming European Clinical Trial Regulation should be a major determinant regarding this issue, particularly via the risk-proportionate approach.

Studies involving drug therapy faced supplementary delays of implementation. Indeed, the complexity introduced by the pharmaceutical issues in the implementation of these studies should not be neglected [5, 37, 48]. However, this complexity is often unanticipated and impacts both, the investigators and pharmacists [25]. Indeed, due to the underestimation of pharmaceutical issues, there is often a delayed involvement of pharmacists in trial implementation [3, 5, 48]. The logistical issues associated with the supply of an appropriate drug formulation with regards to age and route of administration of drug therapy, and/or of placebo, are frequently underestimated, and can result in unexpected costs and delays [3, 32, 48–51]. In our study, one study out of three faced age-related drug formulation issues, and an age-inappropriate formulation led to significantly increased implementation duration. Thus, because it is not unusual and can increase the implementation duration, the need of an age-appropriate formulation, as well as the need for a placebo, should be underscored. The establishment of a relationship between the pharmaceutical companies and the public sector, specifically in the context of the pediatric research, is not always possible [49], particularly due to the lack of financial rewards for the pharmaceutical industry [6, 23]. As a consequence, the pharmaceutical burden can be increased, and if it is not anticipated, delays and financial needs might increase in such a way that it could strongly hamper the course of the study [37].

### Meaning of the study results and implications for policymakers

This study identifies and characterizes issues that may be generalizable on a global scale, and would therefore be of value to the academic pediatric research community at large.

Our findings show that the implementation of interventional hospital-based studies with government funding face the most delays.

With regards to interventional studies involving drug therapy, public-private partnerships appear valuable. However, pharmaceutical companies are not always responsive to requests’ partnerships, mainly due to the lack of return on investment [6, 23]. Academic stakeholders must then grapple with the complexity of pediatrics regulations, which are actually targeted at the pharmaceutical industries. They then face the methodological, ethical, and technical requirements of pediatric clinical trials, often with limited financial resources [5, 38]. The upcoming European Clinical Trial Regulation should be a major determinant regarding this issue, particularly via the risk-proportionate approach.

Finally, documentation of pharmaceutical feasibility should be a prerequisite to any grant allocation. The difficulties in the supply of drugs and placebo in age-appropriate dosages and appropriate route of administration formulations should be particularly evaluated. This measure would ensure adequate funding for the pharmaceutical component and would avoid allocating grant funding to projects that eventually face insurmountable pharmaceutical obstacles. Raising awareness for stakeholders around this topic, as well as the development and validation of pharmaceutical feasibility evaluation tools, would be a judicious axis for improvement [48].

## Conclusion

More pediatric hospital-based studies are needed to address the lack of pediatric research. However, the implementation of such studies faces delays, mainly due to regulatory and pharmaceutical issues. Regarding clinical trials, these delays have the potential to decrease with the upcoming European Clinical Trial Regulation. Although the involvement of the pharmaceutical industry usually reduces implementation delays, such collaborations are not always possible. In addition, difficulties in the supply of drugs and placebo in age-appropriate dosages and appropriate route of administration formulations result in delayed studies. Pharmaceutical issues should, therefore, be anticipated earlier. We encourage the development of a “Research Pharmacist” specifically trained in providing early pharmaceutical expertise, and in developing recommendations consistent with the methodological constraints of studies.

## Additional file

Additional file 1: French hospital-based Biomedical Research System. (DOCX 14 kb)
