# Supplementary material for: Academic pediatric clinical research: factors associated with study implementation duration
Source: BMC Med Res Methodol. 2016 Mar 29;16:36. doi: 10.1186/s12874-016-0138-y (PMC4812626; doi:10.1186/s12874-016-0138-y)
Supplement: Additional file 1: — French hospital-based Biomedical Research System. (DOCX 14 kb) [file 12874_2016_138_MOESM1_ESM.docx]

**Additional file 1.** French hospital-based Biomedical Research System

The French hospital-based research system has four main components:

- The DIRCs (Délégation Interrégionale à la Recherche Clinique): sponsor hospital-based clinical research and manage administrative, legal and financial issues. Every French University hospital is affiliated with one of the seven DIRCs. The DIRC for the Ile-de-France region includes a vast division, the AP-HP (Assistance Publique - Hôpitaux de Paris) located in the Paris conurbation. This study focused on AP-HP sponsored research.

- The Clinical Trial Units are under the authority of the DIRCS. Their main roles are to manage methodological and biostatistical issues, contribute to manage legal issues, and monitor the studies.

- The ANSM (Agence National de Sécurité du Médicament et des Produits de Santé) is a national institution under the authority of the Ministry of Health. It is the delegated health authority; the component authority for French biomedical research involving drug therapy; and the French registration authority for all products and techniques used in biomedical research.

- Central and/or local hospital pharmacies are in charge of the pharmaceutical process. Central hospital pharmacies are specific entities, of which each is affiliated with a public hospital network.
